# The impact of language context on inter-brain synchrony in bilingual families

**DOI:** 10.3389/fcogn.2025.1695132

**Published:** 2026-02-18

**Authors:** Efstratia Papoutselou, Nivetha Saravanan, Guangting Mai, Samantha Harrison, Hilal Dogan Sezer, Douglas Hartley

**Affiliations:** 1 Hearing Sciences, NIHR Nottingham Biomedical Research Centre, Nottingham, United Kingdom; 2 Otology & Hearing Group, Division of Clinical Neuroscience, School of Medicine, University of Nottingham, Nottingham, United Kingdom; 3 MRC Cognition and Brain Sciences Unit, University of Cambridge, Cambridge, United Kingdom; 4 Division of Psychology and Language Sciences, University College London, London, United Kingdom; 5 Nottingham University Hospitals National Health Service (NHS) Trust, Queen's Medical Centre, Nottingham, United Kingdom

**Keywords:** parent–child interaction, bilingualism, neural synchrony, hyperscanning, fNIRS, social neuroscience, second language communication

## Abstract

**Background:**

Bilingualism is increasingly common in families worldwide, yet bilingual individuals remain underrepresented in developmental neuroscience research. In simultaneous bilingualism, children typically acquire two languages simultaneously from birth, while their parents tend to learn the societal language later in life. These differences in language acquisition may influence how parents and children communicate, particularly when interacting in a second language. Neural synchrony, the temporal alignment of brain activity between individuals, has emerged as a key mechanism underlying social connection, communication, and learning in early development. However, little is known about how language choice affects neural synchrony in bilingual parent–child interactions.

**Methods:**

This study used functional near-infrared spectroscopy (fNIRS) hyperscanning to simultaneously record brain activity from 15 bilingual mother–child dyads during naturalistic play. Each dyad completed three conditions: collaborative play in the mother's native language, collaborative play in English (the mother's second language), and independent play. Neural activity was recorded from the prefrontal cortex (PFC) and temporoparietal junction (TPJ), regions associated with social cognition, joint attention, and mentalising. Families took part in a naturalistic free play paradigm, allowing them to interact in a comfortable and ecologically valid manner.

**Results:**

Both native- and English-language play elicited significantly greater neural synchrony across the PFC and the TPJ than independent play, validating the use of naturalistic free play paradigms. No significant overall differences emerged between native and English play, indicating that bilingual dyads maintain inter-brain coupling across languages when both partners are proficient. Exploratory analyses suggested a trend toward higher child-directed synchrony in English play and age-related trends in mother-directed synchrony; however, these effects did not reach statistical significance.

**Discussion:**

Our findings show that bilingualism does not compromise mother–child neural synchrony, supporting the inclusion of linguistically diverse families in developmental neuroscience. They underscore the value of naturalistic paradigms and highlight the need for future research on language proficiency, partner familiarity, and behavioral correlates of synchrony. This work highlights the importance of studying bilingual families in ecologically valid contexts to better understand how language use influences neural coupling in early development.

## 1 Introduction

Bilingualism is a widespread and growing phenomenon, with many families around the world raising children in environments where more than one language is spoken (Byers-Heinlein et al., 2019). In these households, children often grow up managing more than one language and culture from an early age. Despite the prevalence of bilingualism, bilingual individuals, particularly children, are frequently underrepresented in developmental and cognitive neuroscience research unless the study is explicitly focused on bilingualism (Brown et al., 2014; Ransing et al., 2023). This underrepresentation limits our understanding of how bilingualism shapes early cognitive and social development.

Research has shown that bilingual children may follow distinct developmental trajectories in language acquisition and communication compared to their monolingual peers (Byers-Heinlein and Lew-Williams, 2013; Hoff et al., 2012; Soto-Corominas et al., 2020). Simultaneous bilingualism, where children are typically exposed to two languages from birth, often leads to the development of both languages in parallel, but the pace and pattern of acquisition can vary depending on the quantity and quality of input in each language (Hoff and Core, 2013; Meir, 2018; Paradis, 2023; Paradis and Jia, 2016). However, when considering their total conceptual vocabulary -the combined set of unique concepts known across both languages—they often match or exceed their monolingual peers (Barac and Bialystok, 2012; Muszyńska et al., 2025). Bilingual children also tend to demonstrate enhanced metalinguistic awareness, greater sensitivity to social and contextual cues, and advantages in executive functioning (Agostini et al., 2025; Bialystok, 2015; Carlson and Meltzoff, 2008; Romero et al., 2024; Poulin-Dubois et al., 2011). These developmental differences are not deficits but reflect the adaptive strategies bilingual children use to navigate complex linguistic environments.

In contrast, parents in bilingual families might have acquired the societal language (e.g., English) later in life, often during adolescence or adulthood. Even when adults achieve high levels of proficiency in a second language (L2), their use of that language can differ from their native language (L1) in subtle but important ways. Research shows that L2 speakers may exhibit reduced fluency, slower speech rates, and more frequent pauses or self-corrections, especially in emotionally charged or cognitively demanding situations (Abdi Tabari et al., 2024; Feng, 2022; Thoma, 2021). Additionally, second language use can affect emotional expression and nuance; L2 speakers often report a sense of emotional distancing when using their non-native language, which may influence how they express affection, discipline, or empathy in parent-child interactions (Colbeck and Bowers, 2012; Costa et al., 2014; Dylman and Bjärtå, 2019; Naranowicz et al., 2022; Pavlenko, 2012; Sharif and Malik, 2023). These differences may also extend to pragmatic aspects of communication, such as turn-taking, gesture use, and culturally embedded expressions (Emir Özder et al., 2023; Verga and Kotz, 2013). Consequently, even highly proficient bilingual parents may experience small but meaningful changes in how naturally and effectively they communicate with their children in their second language.

Critically, these parental differences in L2 communication are met by distinct processing strategies in the bilingual child. Although children in our sample are native in both languages, their experience of emotion and social signals may still vary based on the language used. For instance, children show greater pupil dilation and sensitivity to emotion words presented in their L1 compared to L2, suggesting L1 facilitates more automatic and deeper emotional (Altarriba and Mathis, 1997; Pavlenko, 2005). Similarly, developmental literature indicates that children's processing of non-native speech can be less efficient, requiring additional executive resources to filter and process the input (Conboy and Kuhl, 2011; Kuhl, 1999). Therefore, a communication environment where both partners are operating in a less-than-fully-fluent mode (the parent's L2 fluency cost and the child's differing processing of L2 emotional/social cues) creates a situation where the mechanisms of social coordination may be stressed.

One promising approach to understanding the impact of bilingualism on parent-child communication is the study of neural synchrony, the temporal alignment of brain activity between individuals during shared experiences (Dumas et al., 2010). From a developmental perspective, synchrony (neural, behavioral and physiological) is believed to support early learning and communication by aligning cognitive and affective states between partners (Feldman, 2012, 2017; Leclère et al., 2014).

Theoretical frameworks such as interactive alignment theory (Pickering and Garrod, 2004) and shared intentionality (Tomasello et al., 2005) suggest that successful communication relies on this alignment, which may scaffold language development, social bonding, and the acquisition of cultural norms. Empirical research supports this view: parent–infant synchrony has been linked to the development of self-regulation, symbolic communication, and empathy across childhood and adolescence (Levy et al., 2019; Busuito et al., 2019; Feldman, 2007b). Synchrony is thought to provide a regulatory function for the infant's emerging neurobiological systems, including stress response, emotional processing, and social cognition (Feldman, 2010, 2007a; Levy et al., 2017, 2021). For example, synchronous interactions have been associated with increased oxytocin release and reduced cortisol levels, suggesting a biological basis for the calming and bonding effects of coordinated engagement (Feldman, 2015).

Neural synchrony has been observed in a variety of social contexts, including conversation, joint attention, and cooperative tasks [reviewed in Carollo and Esposito (2024); Hamilton (2021)]. Within the parent-child hyperscanning literature, the study of neural synchrony has been instrumental in identifying the neural substrates of effective social interaction. In parent–child interactions, higher levels of neural synchrony have been associated with greater emotional attunement, mutual engagement, and successful communication (Deng et al., 2024; Hoyniak et al., 2021; Nguyen et al., 2021b; Papoutselou et al., 2024; Xu et al., 2025; Miller et al., 2019). Studies also consistently show that synchronized activity in the prefrontal cortex (PFC) predicts higher levels of parental sensitivity and child engagement (Azhari et al., 2019; Reindl et al., 2018). More specifically, fNIRS hyperscanning in mother-child dyads during toy play has demonstrated that moment-to-moment synchrony is positively correlated with shared eye gaze and successful verbal turn-taking, highlighting its role as a key mechanism for real-time interactional success (Leong et al., 2017; Nguyen et al., 2021b). Furthermore, the quality of the relationship modulates synchrony, with secure attachments linked to stronger coupling in frontotemporal areas (Birk et al., 2022).

Neural synchrony may also serve as a mechanism for social learning, allowing children to better interpret and internalize the intentions, emotions, and behaviors of their caregivers (Ilyka et al., 2021). This alignment is dynamic, shifting between moments of synchrony and asynchrony, and is influenced by the quality of the interaction, including eye contact, affective mirroring, and turn-taking (Schilbach and Redcay, 2025). Importantly, disruptions to synchrony, whether due to stress, language barriers, or mismatched communication styles, can impact the development of secure attachment, emotional regulation, and social competence (Azhari et al., 2019; Birk et al., 2022).

In this study, we investigated whether the language used during mother–child interactions affect neural synchrony in bilingual families. Specifically, we compared synchrony when dyads interacted in the mother's native language vs. her second language, English. We also included an independent play condition to serve as a baseline for non-interactive engagement. Beyond addressing this specific question, we sought to inform methods for including bilingual families in hyperscanning research. If language choice does not significantly affect synchrony, bilingual families should be able to participate in English-based studies. However, if synchrony is stronger in the native language, it may be important to allow families to interact in their preferred language to capture more ecologically valid data.

To explore these questions, we used fNIRS hyperscanning to simultaneously record brain activity from both mothers and children during naturalistic play. Hyperscanning is a relatively recent methodological advancement in social neuroscience that allows researchers to measure brain activity from two or more individuals at the same time (Carollo and Esposito, 2024; Czeszumski et al., 2020; Zhao et al., 2024). This approach enables the study of inter-brain dynamics during real-time social interaction, moving beyond traditional single-brain paradigms that often rely on passive observation or artificial tasks. Crucially, hyperscanning moves beyond measuring static co-activation, i.e., the simple fact that two brains are active in similar regions, to capture the temporal dynamics of this alignment. Studying these moment-to-moment fluctuations allows us to infer how an interactive, coupled system is established and maintained (Czeszumski et al., 2020). It can reveal whether one individual's neural activity leads or follows their partner's, suggesting a system where brains mutually adapt and predict each other's states in real-time. In the context of parent-child interaction, this inter-brain synchrony is thought to be a neural marker of a shared intentional and attentional state, reflecting the dyad's capacity to build a common ground of understanding (Czeszumski et al., 2020).

Hyperscanning has thus opened new avenues for understanding how brains align during communication, cooperation, and emotional exchange, making it particularly well-suited for studying parent–child relationships in ecologically valid settings (Carollo and Esposito, 2024; Czeszumski et al., 2020; Zhao et al., 2024).

Our regions of interest included the prefrontal cortex (PFC) and temporoparietal junction (TPJ), areas implicated in social cognition, joint attention, and mentalising (Monticelli et al., 2021; Nejati et al., 2023; Tei et al., 2017). The PFC is involved in executive functions, emotion regulation, and goal-directed behavior (Friedman and Robbins, 2022), while the TPJ plays a key role in perspective-taking and understanding others' intentions (Krall et al., 2015). These regions have been shown to exhibit inter-brain synchrony during cooperative and communicative tasks [reviewed in Czeszumski et al. (2020)], making them ideal targets for studying the neural dynamics of bilingual parent–child interaction. It is proposed that synchrony in these specific regions is not just a byproduct of a shared environment, but a core mechanism of successful social connection. Specifically, alignment in the TPJ may reflect the real-time, mutual process of inferring and updating beliefs about each other's intentions (i.e., 'Theory of Mind in action'). Simultaneously, synchrony in the PFC may represent the alignment of shared goals and the top-down cognitive scaffolding one partner (likely the parent) provides to guide the interaction and manage joint attention (Czeszumski et al., 2020).

In this study we hypothesize that this neural coupling is sensitive to the relative cognitive load of the communication language. Given our sample (i.e. children are native in both languages and mothers are native in one language and proficient in English) this leads to two competing possibilities. If the mother's English proficiency allows for linguistic processing that is as automatic as her native language, the cognitive load should be minimal in both conditions. In this scenario, we would expect to see similarly robust PFC and TPJ synchrony, as neural resources in both contexts would be freely dedicated to the core social-cognitive processes of aligning intentions and goals. However, if communicating in English, even as a proficient speaker, imposes a higher cognitive load than the automaticity of the native language, this may divert the parent's neural resources toward the demands of language control, retrieval, and encoding. We propose this increased cognitive ‘friction' could then disrupt or reduce the efficiency of the neural coupling required for seamless social alignment, thereby leading to weaker inter-brain synchrony in the English condition.

To ensure ecological validity and to capture the richness of real-world communication, we employed a naturalistic free play paradigm. This approach allowed families to interact in a way that felt comfortable and familiar, without imposing rigid task demands or scripted dialogue. By observing spontaneous, child-directed play in both the native and second language contexts, we aimed to better understand how language use shapes neural synchrony in everyday social interactions.

## 2 Materials and methods

### 2.1 Participants

Fifteen mother–child dyads participated in this study. Mothers had a mean age of 37.95 years (SD =5.30), and all were non-native English speakers who completed an online English language proficiency test from *Transparent Language* (Transparent Language, 2025) that provided a CEFR score; parents who scored below a C1 level were not eligible to participate in the study. Children (6 female) were between the ages of 3 and 4;11 years (mean age = 3.93 years, SD =0.64). All participants had normal or corrected-to-normal vision and no known hearing, language, or cognitive impairments. Children with a history of cognitive or motor difficulties, as reported by their parents, were excluded. Participants were recruited through the National Institute of Health Research Nottingham Biomedical Research Center Hearing Sciences participant database and via online advertisements in parent-focused Facebook groups in the Nottinghamshire area. Ethical approval for this study was granted by the University of Nottingham Faculty of Medicine and Health Sciences Research Ethics Committee.

### 2.2 Paradigm

**Figure 1:**
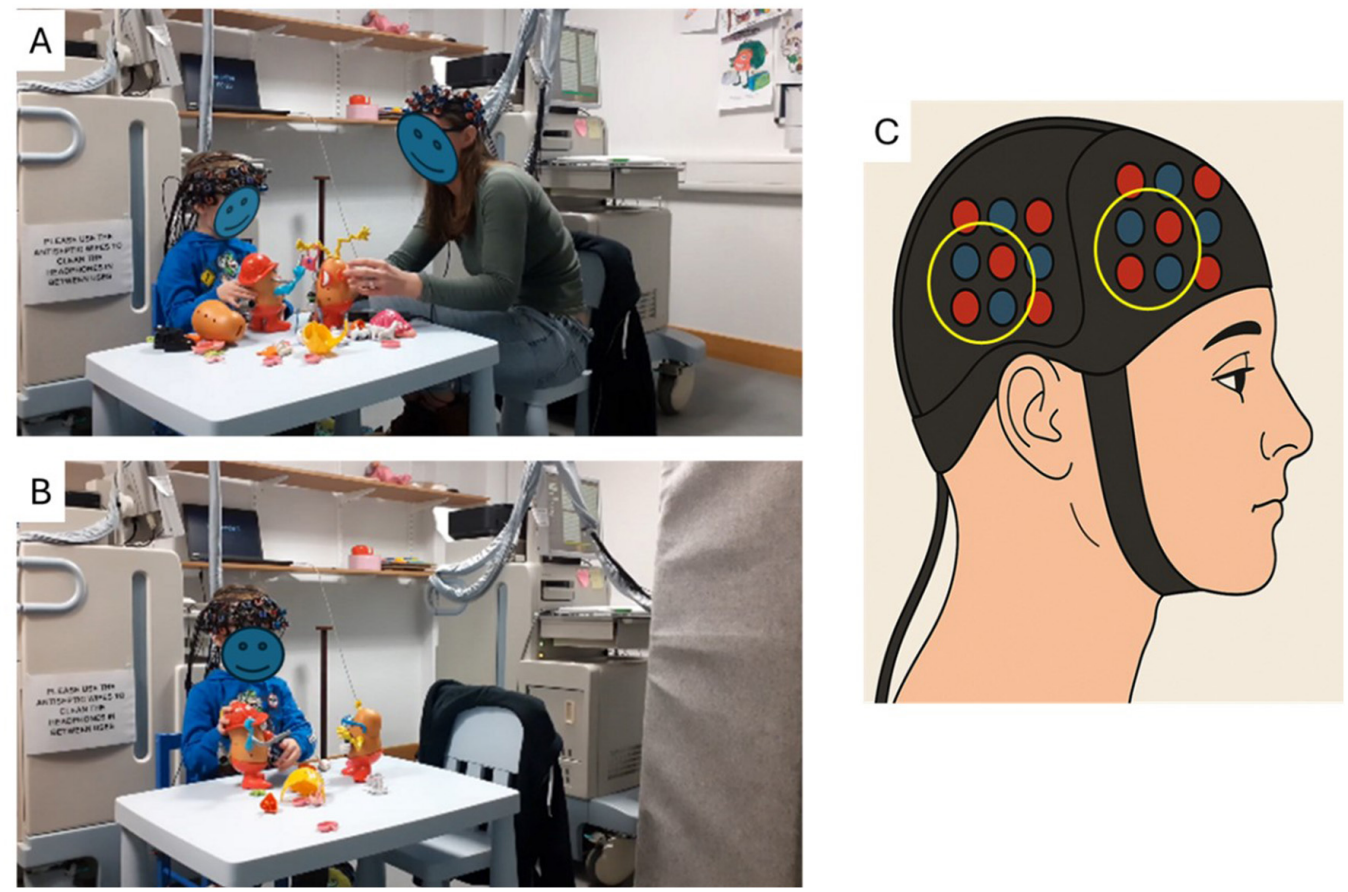
**(A)** Experimental setup during the interactive condition. **(B)** Setup during the independent condition. **(C)** Schematic representation of probe placement over the regions of interest (ROIs), indicated by yellow circles, bilaterally covering the prefrontal cortex and the temporoparietal junction (left hemisphere shown). Red circles denote emitter optodes, while blue circles indicate detector optodes. The illustration is not to scale and is intended for visual reference only.

Each mother–child dyad attended a single research session. Mothers provided written informed consent, and children gave verbal assent. Dyads were seated at a toddler-appropriate table at a 90° angle to one another. Age-appropriate toys, including a Mr. Potato Head set with accessories and building blocks, were provided to facilitate naturalistic play without performance pressure (Figure 1A). The task was explained to the dyads, and they were given time to familiarize themselves with the toys and the fNIRS cap. The cap was placed on the mother first, followed by the child, to minimize the time the child needed to wear it. Researchers ensured correct optode placement and signal quality before beginning the session.

Each session was video recorded using two cameras positioned to capture both participants. The session lasted approximately 45 min and consisted of three conditions: (1) a Native-interactive condition that involved collaborative play in the dyad's native language, (2) an English-interactive condition that involved collaborative play condition in English, and (3) an Independent condition that involved independent play. Each condition lasted 5 min and was repeated twice. During rest periods, dyads were offered a break, and the mother was moved to an adjacent table for the independent condition if an independent play condition was next. The order of conditions was pseudorandomized across dyads.

In the native language collaborative condition, mothers and children were instructed to play together as they would at home, using their shared native language. In the English collaborative condition, the same instruction was given, but dyads were asked to interact exclusively in English. In the independent condition, the mother and child were separated by an opaque screen and played silently with their respective toys (Figure 1B). A researcher remained in the room during this condition to ensure the child's safety.

### 2.3 fNIRS data acquisition

Brain activity from each member of the dyad was recorded using two continuous wave fNIRS systems (Hitachi ETG-4000, Japan), each operating at a sampling rate of 10 Hz. Each participant was connected to a separate system, and both devices were synchronized via a custom MATLAB script that launched data collection simultaneously across the two systems.

The ETG-4000 emits near-infrared light at two wavelengths (695 nm and 830 nm) to monitor changes in concentrations of oxygenated (HbO) and deoxygenated hemoglobin (HbR). The mothers wore caps fitted with 48 optodes arranged in three arrays: a 3 × 5 grid over the prefrontal cortex (PFC) and two 3 × 3 grids over the left and right temporoparietal junctions (TPJ) (Figure 1C). For the children, a lighter cap with 16 optodes was used, arranged in four 2 × 2 arrays targeting the same brain regions. This reduced configuration was chosen to enhance comfort while still capturing activity in the key areas of interest. The spacing between optodes was maintained at 3 cm for both adults and children. To accommodate individual differences in head size, each child's head circumference was measured prior to cap placement. Optode positioning followed the international 10–20 system, with the left TPJ aligned around CP5, the right TPJ around CP6, the left PFC around AF3, and the right PFC around AF4 (Jasper, 1958).

### 2.4 fNIRS data processing

Preprocessing of fNIRS followed the process in our previous publications (Anderson et al., 2017; Lawrence et al., 2018, 2021; Mushtaq et al., 2019; Papoutselou et al., 2024; Wiggins and Hartley, 2015). This was conducted via MATLAB 2019b (MathWorks) using the HOMER2 package (Huppert et al., 2009) incorporating customized scripts. First, the fNIRS intensity was converted into changes in optical density. Second, motion artifacts were corrected using wavelet filtering that removed outlying wavelet coefficients outside the 0.725 inter-quantile range (Molavi and Dumont, 2012) and were bandpass filtered between 0.01 and 0.5 Hz (via a 3rd-order zero-phase Butterworth filter) to attenuate low-frequency drifts and cardiac oscillations. Third, optical density was converted to estimated changes in the concentration of HbO and HbR through the modified Beer-Lambert Law (Huppert et al., 2009). Finally, the haemodynamic modality separation (HMS) algorithm was applied to further extract cortical activities (Yamada et al., 2012). This was done by isolating the functional component of the haemodynamic signals from systemic physiological noise (Yamada et al., 2012) assuming that changes in HbO and HbR are negatively correlated in the functional responses but positively correlated with motion and physiological noises (Yamada et al., 2012). This algorithm has been shown to yield fNIRS responses with more reliable signal quality (Wiggins et al., 2016).

Alongside preprocessing, channels with poor signal quality were also detected. The Scalp Coupling Index (SCI) that effectively identifies poor fNIRS signals was adopted (Lawrence et al., 2018; Mushtaq et al., 2019; Pollonini et al., 2014). The signals with the two wavelengths were first bandpass filtered into 0.5–2.5 Hz that represents the cardiac elements captured by fNIRS and were correlated with each other. Following the criteria used in our previous fNIRS studies (Lawrence et al., 2021; Mushtaq et al., 2019; Papoutselou et al., 2024), the worst 5% of channels (across all participants and sessions) that indicate poor optode contacts were identified and excluded from subsequent analyses.

Furthermore, due to the naturalistic free-play paradigm, we considered that systemic physiological confounds may not be completely eliminated after preprocessing. We thus further divided the preprocessed signals into frequency sub-bands that correspond to specific types of possible physiological confounds and then focused on the band which led to most significant inter-brain synchrony comparing the interactive with the independent play. We considered three sub-bands based on the previous literature: 0.01–0.05 Hz, 0.05–0.2 Hz and 0.2–0.5 Hz which reflect ranges for the autonomic, myogenic and respiratory activities, respectively (Rossi et al., 2007; Rojas et al., 2017). Here, we focused on the sub-band at 0.05–0.2 Hz (via a 3rd-order zero-phase Butterworth filter) that has been shown to most effectively attenuate systemic physiological confounds after preprocessing and result in most significant synchrony observed by our previous hyperscanning study (Papoutselou et al., 2024).

Inter-brain synchrony between the mother-child dyads was then measured using Phase Transfer Entropy (PTE; Lobier et al., 2014; Ursino et al., 2020). PTE calculates the degree of certainty in one signal given the past values of another signal using phase entropies. It thus quantifies the extent to which two different neural activities causally influence one another (Lobier et al., 2014). Compared to other methods typically used in fNIRS hyperscanning studies such as wavelet coherence transformation, PTE allows for measuring the directionality that considers potential time lags between brain activities in each participant and quantifies the degree of inter-brain synchrony in different directionalities (i.e., how the brain signals of one participant follow the other) (Cao et al., 2018; Haresign et al., 2022; Wang and Chen, 2020). We have shown that using PTE can effectively quantify inter-brain synchrony. We applied the open-accessed MATLAB codes that calculate the PTE (Fraschini and Hillebrand, 2017) using the following formulas (Lobier et al., 2014):


$$\begin{array}{r} PTE_{x\to y}=H\left(\theta_{y}\left(t\right),\theta_{y}\left(t^{\prime }\right)\right)+H\left(\theta_{y}\left(t^{\prime }\right),\;\theta_{x}\left(t^{\prime }\right)\right)\\ -\;H\left(\theta_{y}\left(t^{\prime }\right)\right)-\;H\left(\theta_{y}\left(t\right),\theta_{y}\left(t^{\prime }\right),\theta_{x}\left(t^{\prime }\right)\right) \end{array}$$ (1)

$$\begin{array}{l} t^{\prime }=t-\delta \end{array}$$ (2)

$$\begin{array}{l} H=\;\Sigma plog\left(p\right) \end{array}$$ (3)

where *PTE*_*x*→*y*_ (formula (1)) calculates the synchrony between signals *x(t)* and *y(t)* with information flowing from *x* to *y* (i.e., *x* leads *y*). θ_*x*_*(t)* and θ_*y*_*(t)* refer to the instantaneous phase (via Hilbert transform) at time *t* of the two signals, respectively. δ (formula (2)) denotes the time lag between the two signals. *p* refers to the probability of instantaneous phases and *H* is the phase entropy (formula (3)). Greater *PTE*_*x*→*y*_ reflects smaller entropy (i.e., greater certainty) of θ_*y*_ given in the past values of θ_*x*_, hence informing greater information flows from *x* to *y*. Here, PTE values were measured with different δ at steps of 0.5 s and were then summed across 0.5–5 s. This is according to previous reports which showed that inter-brain synchrony between adults and children peaked when the time lag at ~4 s (Piazza et al., 2020; Zhao et al., 2021). The pre-processed signals were averaged across channels within each ROI in the time domain before PTE was applied (Papoutselou et al., 2024). As there were four ROIs (bilateral TPJ and bilateral prefrontal cortices) for both the child and mother, this resulted in 16 PTE values for each directionality (child leading mother or mother leading child) for each dyad. In this context “mother-directed” synchrony refers to instances where the mother's neural activity leads or initiates the directional influence on the child's brain signals, whereas “child-directed” synchrony refers to cases where the child's neural activity leads or drives the interaction.

### 2.5 Statistical analysis

All analyses were conducted using SPSS, with the significance threshold set at p < 0.05 (two-tailed). Effect sizes and 95% confidence intervals (CIs) were reported where appropriate. Given the small sample size (*N* = 15), the assumption of normality was evaluated using the Shapiro–Wilk test and Greenhouse–Geisser corrections were applied when assumptions of sphericity were violated.

To examine effects of experimental manipulations and neuroanatomical specificity, we conducted a repeated-measures ANOVA including the within-subjects factors condition (3 levels: English interactive, Native interactive, independent play), directionality (2 levels: mother-directed vs. child-directed), child brain region (4 levels: left TPJ, right TPJ, left PFC, right PFC), and mother brain region (4 levels: left TPJ, right TPJ, left PFC, right PFC). This model allowed us to assess whether neural synchrony differed across conditions, whether effects varied according to the direction of connectivity, and whether synchrony patterns were shaped by the specific child and mother brain regions involved.

Follow-up analyses focused on main effects and interactions identified in the model. Pairwise comparisons with Bonferroni correction were used to examine differences between conditions, as well as differences across child and mother brain regions. Where a significant interaction was detected, simple effects analyses were conducted. For interpretability, higher-order interactions that were non-significant were not pursued further, and subsequent analyses were collapsed across those factors. Where collapsing across factors was applied, this is explicitly indicated in the Results section.

To determine whether the observed neural synchrony exceeded what would be expected by chance, we conducted a non-parametric pairwise sign-flip permutation test (Nichols and Holmes, 2002). This approach preserves the dependency structure within each dyad while generating a null distribution in which any systematic relationship between conditions is eliminated. For each comparison, we randomly flipped the sign of the within-pair difference between conditions (e.g., Interactive – Independent) 10,000 times, recomputed the mean difference for each permutation, and used the resulting null distribution to estimate a two-tailed *p*-value. This procedure tests whether the observed group-level difference could plausibly arise from data in which no condition-specific synchrony is present and therefore provides a stringent control against spurious synchrony emerging from shared environment, parallel task timing, or analytic bias.

Pearson correlation analyses were conducted to assess the relationship between maternal age and child age with neural synchrony across conditions and directions. Correlation coefficients (*r*) and significance values were reported, with emphasis on both statistically significant and marginal trends.

## 3 Results

The repeated-measures ANOVA including condition, directionality, child brain region, and mother brain region revealed several significant main effects. There was a main effect of condition (*F*_(2, 28)_ = 4.57, *p* = 0.019), indicating that neural synchrony differed across the three play contexts. A main effect of child brain region was also observed (*F*_(3, 42)_ = 6.91, *p* < 0.001), showing that synchrony varied depending on which child region was involved. Similarly, a main effect of mother brain region (*F*_(3, 42)_ = 14.15, *p* < 0.001) indicated that synchrony also differed according to the maternal region. In contrast, there was no main effect of directionality (*F*_(1, 14)_ = 0.06, *p* = 0.806).

Among the interactions, only the directionality × mother brain region interaction was significant (*F*_(3, 42)_ = 4.16, *p* = 0.011). All other two-way, three-way, and four-way interactions were non-significant indicating that the effects were largely independent of higher-order combinations of factors (Condition × Directionality: *F*_(2, 28)_ = 0.489, *p* = 0.619; Condition × Child Region: *F*_(6, 84)_ = 1.216, *p* = 0.306; Condition × Mother Region interaction, *F*_(6, 84)_ = 0.953, *p* = 0.464; Child Region × Mother Region: *F*_(9, 126)_ = 1.005, *p* = 0.440; Condition × Directionality × Child Region, *F*_(6, 84)_ = 0.688, *p* = 0.661; Condition × Directionality × Mother Region, *F*_(6, 84)_ = 0.568, *p* = 0.755; Condition × Child Region × Mother Region, *F*_(18, 252)_ = 1.097, *p* = 0.358; Directionality × Child Region × Mother Region, *F*_(9, 126)_ = 1.810, *p* = 0.088; Condition × Directionality × Child Region × Mother Region: *F*_(18, 252)_ = 1.354, *p* = 0.161).

### 3.1 Effect of condition on neural synchrony

Given that the repeated-measures ANOVA revealed a significant main effect of condition, we conducted simple effects analyses to examine differences between individual conditions. Pairwise comparisons with Bonferroni correction indicated that there were no significant differences between the English interactive and independent play (mean dif. = −0.021, *p* = 0.096), between the Native interactive and independent play (mean dif. = −0.009, *p* = 0.421), or between English interactive and Native interactive conditions (mean dif. = 0.012, *p* = 0.191). Estimated marginal means suggested that the interactive conditions showed numerically higher synchrony than independent play.

To increase sensitivity in comparing interactive vs. independent play, and because no significant difference was observed between English and Native interactive conditions, the two interactive conditions were averaged. This comparison should be considered exploratory.

**Figure 2:**
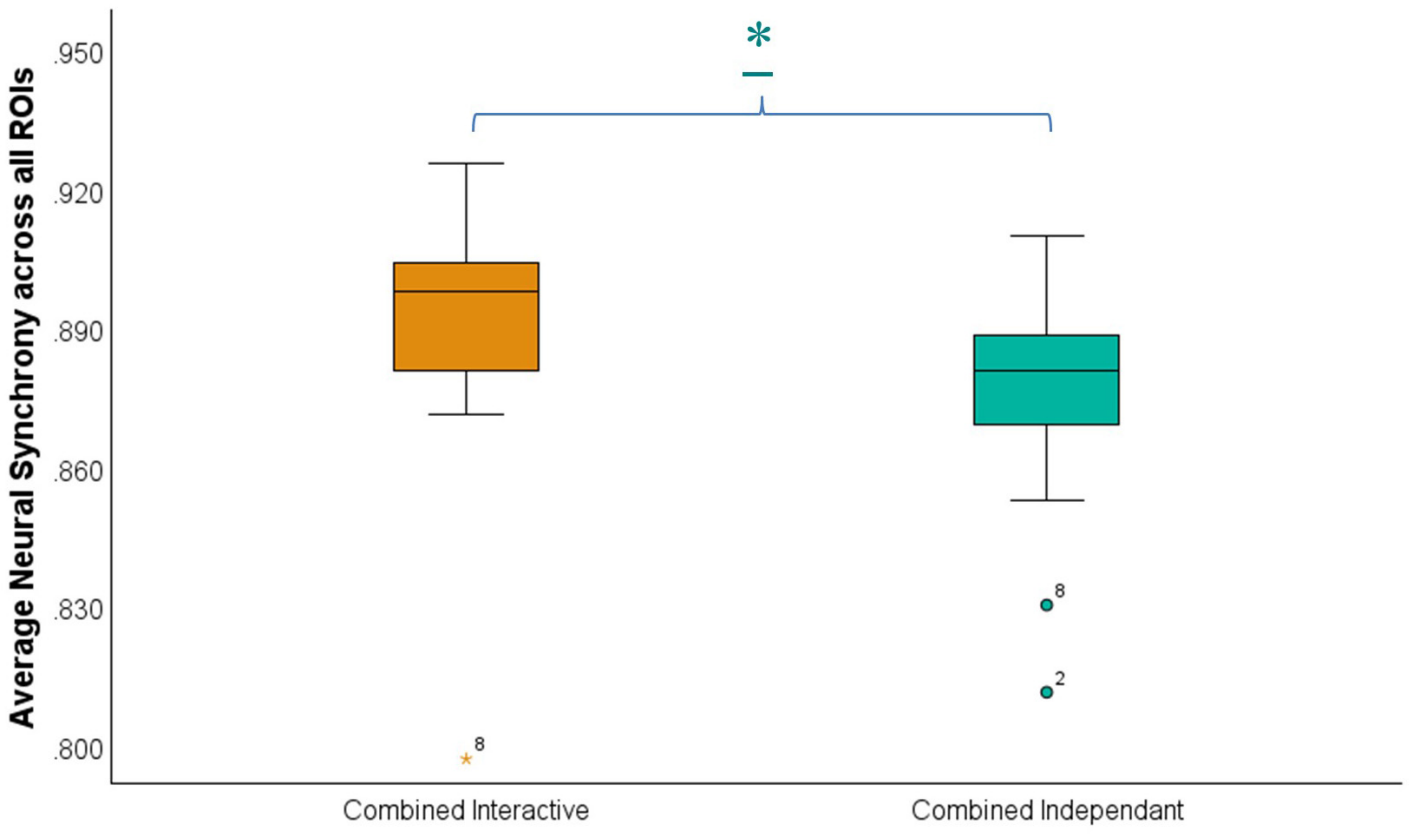
Boxplots of mean neural synchrony across all regions of interest (ROIs) during interactive and independent conditions. Bars represent the mean synchrony values for parent-led and child-led interactions. Neural synchrony was significantly greater in the interactive conditions compared to the independent condition (*t*([14] = −2.207, *p* = 0.022).

A paired *t*-test was conducted and showed that the average neural synchrony during the interactive play M = 0.89, SD = 0.029) was significantly higher compared to the independent play (M = 0.87, SD = 0.026), (*t*([14]= −2.207, *p* = 0.022) (Figure 2). The effect size, calculated as Cohen's d, was 0.57 (95% CI [0.014, 1.109]), indicating a medium effect.

To validate that the difference between interactive and independent play reflected genuine condition-specific synchrony rather than chance similarity, we performed a sign-flip permutation test on the combined child- and parent-directed synchrony values. The observed mean synchrony difference (Interactive > Independent) was 0.0146. Across 10,000 permutations, only 3.34% of permuted datasets produced a difference of this magnitude or greater, yielding a *p* = 0.0334. This indicates that neural synchrony during interactive play was significantly greater than expected by chance, confirming that the observed effect reflects meaningful interpersonal coupling rather than general co-activation.

### 3.2 Effect of region on neural synchrony during interactive conditions

Follow-up pairwise comparisons for the significant main effect of child region showed that neural synchrony was greater in the left PFC compared with the left TPJ (mean dif. = 0.015, *p* = 0.029) and. No significant differences were observed between the right TPJ (mean dif. = 0.014, *p* = 0.066) and the left PFC and right PFC (*p* = 0.962), or between TPJ regions and the right PFC (all *p* = 1.000).

For the main effect of mother region, pairwise comparisons revealed that both the left and right PFC showed significantly greater synchrony than the left TPJ (left PFC vs. left TPJ: mean dif. = 0.059, *p* < 0.001; right PFC vs. left TPJ: mean dif. = 0.051, *p* = 0.036) and the right TPJ (left PFC vs. right TPJ: mean dif. = 0.056, *p* = 0.004; right PFC vs. right TPJ: mean dif. = 0.051, *p* = 0.036). No significant differences emerged between the two PFC regions (*p* = 1.000) or between the TPJ regions (*p* = 1.000). All *p*-values were Bonferroni corrected over the number of regions (i.e., four) for both directionalities.

Taken together, these results indicate that synchrony was particularly pronounced involving prefrontal regions of both children and mothers, whereas synchrony was significantly lower while regions of TPJ were involved.

### 3.3 Effect of language on neural synchrony

Despite the lack of significant interaction between condition and directionality, we conducted exploratory pairwise comparisons to examine whether synchrony varied between conditions for respective directionalities. These analyses were motivated by our a priori interest in the differences between the English interactive and Native interactive conditions and how they may differ during child-directed and mother-directed interactions, respectively.

We performed an exploratory *post hoc* analysis for the interaction between condition and direction (even though it was not statistically significant). Paired *t*-tests showed that child-directed and mother-directed neural synchrony did not differ statistically in either language condition (English: mean dif. = 0.013, *p* = 0.373; Native: mean dif. = −0.008, *p* = 0.458).

**Figure 3:**
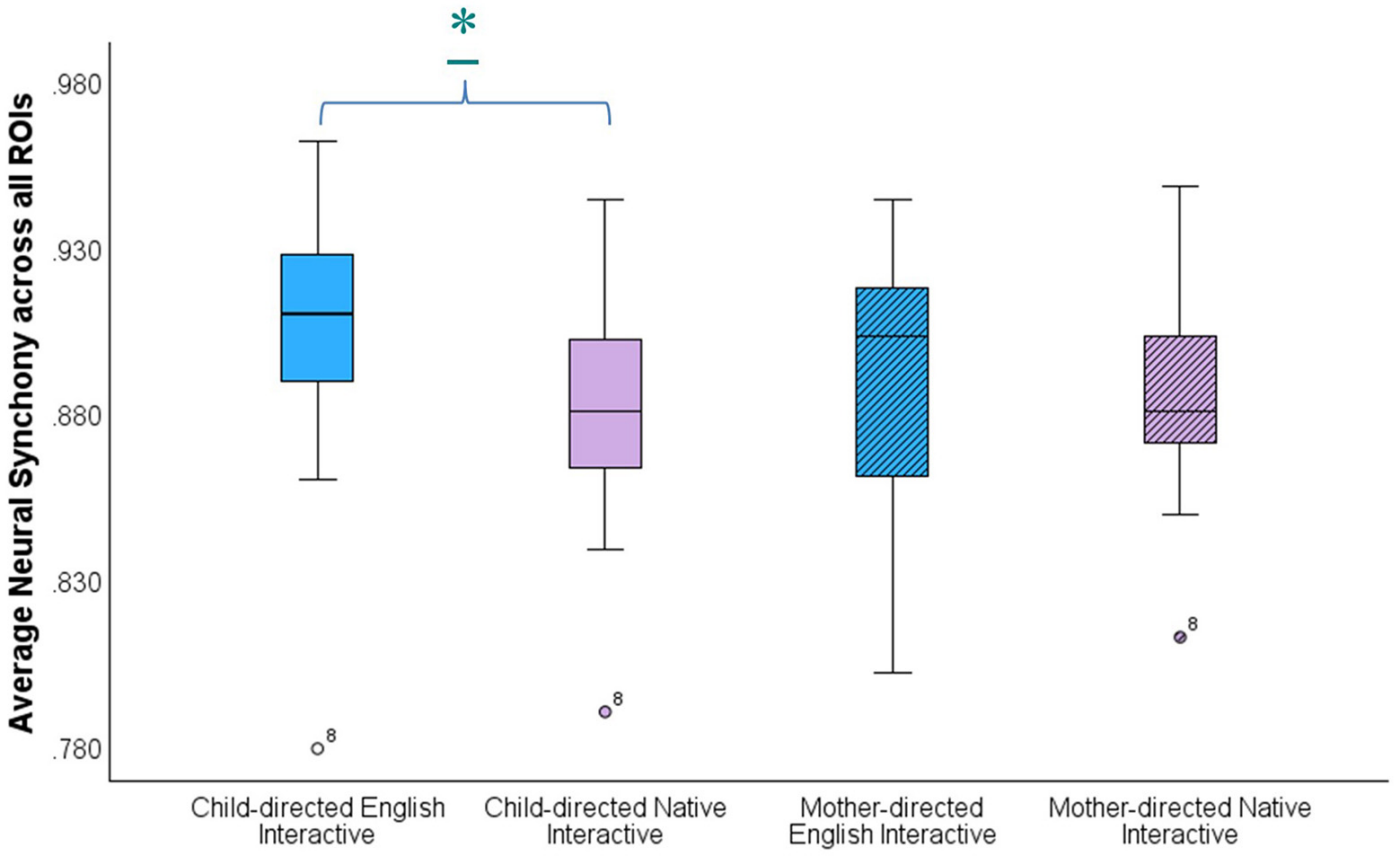
Boxplots of mean neural synchrony across all regions of interest (ROIs) during the interactive conditions. Bars represent the mean synchrony values for child-directed and mother-directed synchrony in the English (blue) and native (purple) interactive play. Neural synchrony was significantly greater in the child directed English vs. the child directed native play (mean dif. = 0.023, *p* = 0.026).

However, when comparing neural synchrony in each condition for each direction, child-directed neural synchrony was significantly higher in the English compared to the Native condition (mean dif. = 0.023, *p* = 0.026). There was no statistically significant difference between language conditions for the mother-directed neural synchrony (mean dif. = 0.001, *p* = 0.910) (Figure 3). A corresponding permutation test compared the two interactive language conditions. The observed difference (English > Native) was 0.0119. Under the null distribution generated from 10,000 sign-flip permutations, this effect did not exceed the threshold for statistical significance (*p* = 0.0574). This aligns with the ANOVA findings, indicating that neural synchrony did not reliably differ between the English and native-language interactive contexts.

### 3.4 Effect of age on neural synchrony

**Figure 4:**
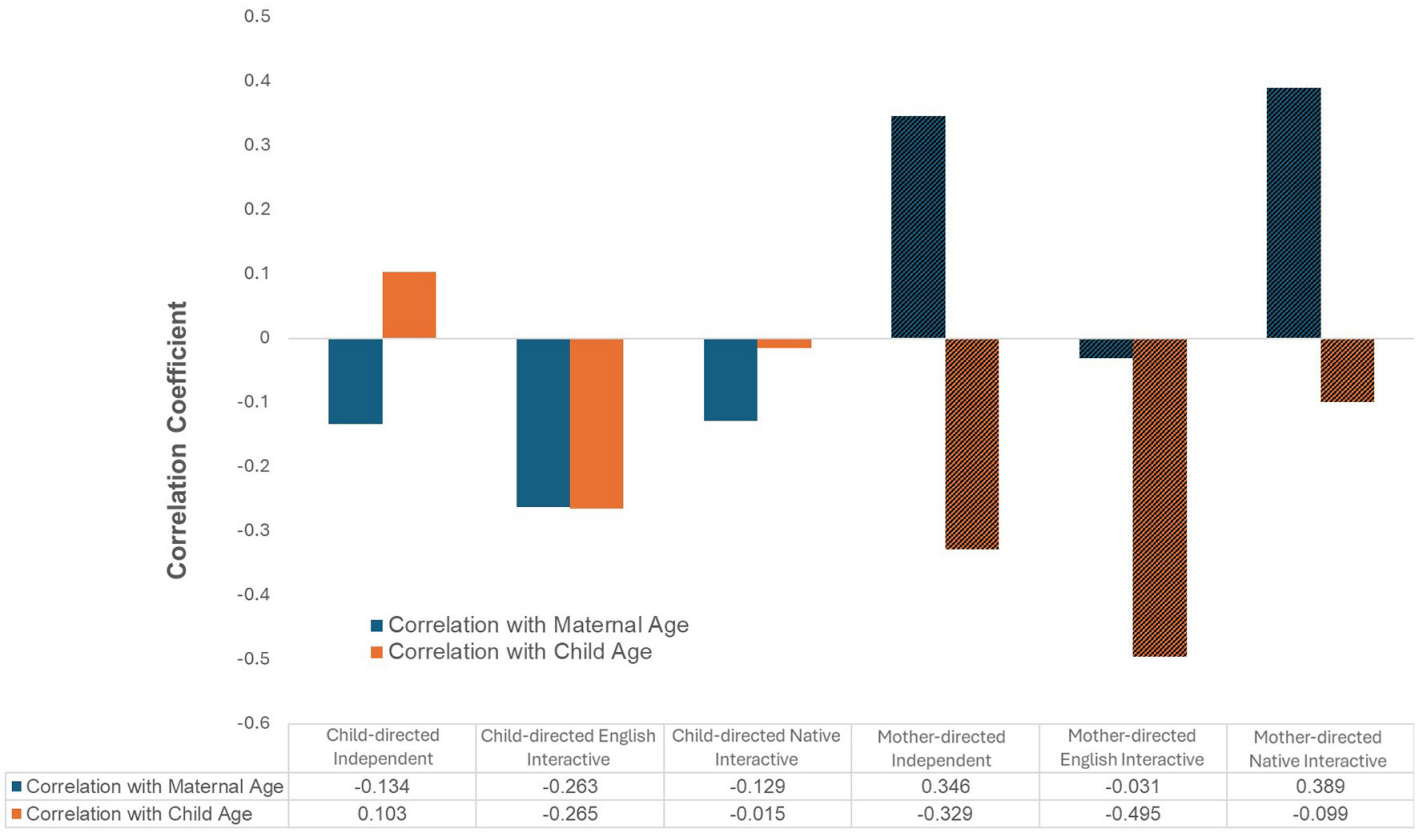
Bar chart displaying Pearson correlation coefficients between neural synchrony across conditions and direction with maternal and child age. Each bar represents the strength and direction of the correlation between age and a specific variable. Positive values indicate a direct relationship, while negative values indicate an inverse relationship.

A Pearson correlation was conducted to examine the relationship between the mothers' age and the neural synchrony across conditions and directions. No comparison reached the level for statistical significance. Similarly, there was no correlation between the child's age and neural synchrony. Some correlations were of moderate magnitude but did not meet conventional thresholds for significance, and are therefore reported for descriptive purposes only. There was a negative correlation between the child's age, and the mother directed neural synchrony during the English-interactive condition (*r*_(14)_ = −0.495, *p* = 0.061) as well as a positive correlation between the mother's age and the mother directed neural synchrony during the Native-interactive condition (*r*_(14)_ = 0.389, *p* = 0.152). Lastly, mother directed neural synchrony during the independent condition was positively correlated with maternal age (*r*_(14)_ = 0.346, *p* = 0.206) and negatively correlated with child age (*r*_(14)_ = −0.329, *p* = 0.1231) (Figure 4).

## 4 Discussion

This study provides evidence that bilingual mother–child dyads exhibit robust neural synchrony during naturalistic interactive play, regardless of the language used. The permutation-based validation further demonstrated that the synchrony observed during interactive play was significantly greater than would be expected from temporally unrelated or random data, confirming that the coupling we report reflects genuine interpersonal alignment rather than an artifact of shared task structure or analytic bias. The finding that interactive play elicited significantly higher neural synchrony than independent play validates the use of a free play paradigm in developmental neuroscience (similar to Papoutselou et al., 2024). This has important implications for research design, particularly in clinical and applied settings where rigid, structured paradigms may not reflect the natural dynamics of parent–child interaction.

Clinical guidelines often recommend allowing families to interact in environments and languages that feel most natural to them (Finders et al., 2023; Rosales et al., 2023), and our findings support this approach by showing that meaningful neural coupling can be captured in ecologically valid, less stringent contexts. However, it is important to note that these conclusions are specific to the current experimental design, which involved a naturalistic and unstructured play condition between mothers and children. They may not necessarily generalize to more structured testing paradigms that emphasize verbal communication, where differences in interbrain synchrony could emerge not only from parental familiarity with the language but also from inherent differences between the languages themselves.

Our regional analyses revealed a prefrontal advantage for both partners with higher cross-brain synchrony observed in PFC than TPJ across all conditions. Importantly, these effects emerged as main effects in the full model and were not moderated by condition, indicating that the PFC predominance represents a general feature of parent–child coupling rather than a task-specific phenomenon. Nonetheless, this aligns with hyperscanning studies showing that cooperative engagement consistently elicits inter-brain coupling in lateral and frontopolar PFC, supporting shared attention and executive control (Cui et al., 2012; Liu et al., 2016; Reindl et al., 2018). In contrast, lower TPJ synchrony fits with accounts that mentalising regions are recruited more intermittently during naturalistic play, where continuous coordination dominates (Redcay and Schilbach, 2019). Parent–child PFC synchrony has also been linked to relationship quality and real-time behavioral alignment (Azhari et al., 2019), and micro-dynamics such as gaze and affective contingencies further strengthen this coupling (Leong et al., 2017; Piazza et al., 2020).

Finally, the prominence of PFC in bilingual dyads may reflect bilingualism's enhancement of domain-general control, consistent with meta-analytic evidence identifying PFC as a core hub for cooperative synchrony (Czeszumski et al., 2020). Specifically, the bilingual experience is known to increase the engagement and specialization of left frontal regions (such as the Inferior Frontal Gyrus, and the PFC) for both language control and attentional tasks in children and adults (Arredondo et al., 2019, 2022). This PFC synchrony, indicative of shared executive function, suggests that the mother's high English proficiency may have effectively neutralized the potential ‘cost' of second language processing, allowing the executive control benefits of bilingualism to support inter-brain alignment equally well in both conditions (Bialystok, 2011). It is important however to emphasize that this study was not sufficiently powered to detect in depth regional differences and further work is needed to determine whether these patterns are specific to interactive contexts or generalize more broadly.

Importantly, we found no significant difference in neural synchrony between the native language and English conditions in our cohort of mother and 3- to 5-year-old children. Permutation-based analyses indicated that the difference in neural synchrony between the English and Native language interactive conditions did not exceed what would be expected by chance, suggesting that language context, in this highly proficient bilingual sample, does not meaningfully modulate inter-brain coupling. This aligns with developmental literature showing that by age three, early bilingual children often demonstrate strong receptive and expressive skills in both languages, as well as flexible language switching (Bialystok, 2017; Byers-Heinlein and Lew-Williams, 2013; Hoff and Core, 2013), (Carlson and Meltzoff, 2008). Moreover, bilingual children have been shown to perform similarly to monolingual peers in social interaction tasks when they are proficient in both languages (Beauchamp et al., 2023), suggesting that language background may not hinder social engagement in familiar contexts.

Furthermore, bilingualism has been associated with enhanced executive functioning, cognitive flexibility, and perspective-taking, which may support children's ability to engage meaningfully across languages (Gordon, 2016; Bialystok, 2011; Yurtsever et al., 2023; Carlson and Meltzoff, 2008). These socio-cognitive strengths could help maintain synchrony even when one language is less frequently used or less proficiently spoken. Additionally, bilingual families often develop adaptive communication strategies, including increased reliance on non-verbal cues and contextual scaffolding, which may buffer against potential linguistic mismatches and support synchrony (Rochanavibhata et al., 2023; Tare and Gelman, 2011).

Our exploratory *post hoc* analysis revealed a nuanced finding: while overall synchrony did not differ by condition or directionality, child-directed synchrony was significantly higher in the English play condition compared to the native language condition. This may reflect the child's ability to engage the mother may be more sensitive to language context (i.e. when communicating in English). The children in our cohort were attending English-speaking nurseries and may have been involved more frequently in English-language play. Another possible explanation is that children may be more active initiators in English contexts, producing clearer or more directive signals that elicit stronger neural coupling from the mother. Interestingly, mother-directed synchrony did not differ significantly across conditions, suggesting that the mother's responsiveness may be less sensitive to language context than the child's ability to drive synchrony. However, this analysis was not statistically significant and given our relatively modest sample size further work would be needed to validate this finding.

We also explored correlations between age and neural synchrony. While the comparisons did not reach statistical significance, two trends are worth noting. First, there was a moderate negative correlation between the child's age and mother-directed neural synchrony during collaborative play in English. This may suggest that younger children rely more heavily on their parents to guide and scaffold interactions, particularly in a second-language context. As children grow older and become more autonomous communicators, the need for parental scaffolding may diminish, leading to reduced mother-directed synchrony. Second, there was a moderate positive correlation between maternal age and mother-directed synchrony during native language play, and a similar trend during independent play. These findings may reflect age-related differences in maternal interaction style, with older mothers potentially engaging in more structured or attuned interactions. Conversely, the moderate negative correlation between child age and mother-directed synchrony during independent play may again reflect increasing child autonomy with age.

### 4.1 Implications, limitations, and future directions

Traditionally, developmental neuroscience studies have favored monolingual English-speaking families to avoid confounding effects of second language processing. However, our findings challenge this exclusionary practice. We demonstrate that in families where both parent and child are bilingual from early life and the parent is proficient in English, language background does not compromise the integrity of neural synchrony. This underscores the importance of including linguistically diverse families in social neuroscience research, especially as bilingualism becomes increasingly prevalent worldwide.

Future research should explore families with varying degrees of language proficiency, including those where the parent is less fluent in the second language or where the child is not bilingual from birth. Prior work has shown that factors surrounding the timing and context of second language acquisition can significantly influence children's ability to interact and communicate (Hartshorne et al., 2018; Paradis, 2023; Qiao, 2024), making it imperative to also investigate how these variables shape communicative outcomes within bilingual families.

A further limitation concerns the breadth and reliability of the language measures collected. Although mothers were asked to complete an at-home proficiency assessment in English, these measures were not supervised and did not capture the full complexity of bilingual experience, such as language dominance, frequency of use, or the child's exposure across contexts. The purpose of this preliminary study was not to comprehensively characterize the families' linguistic backgrounds, but rather to explore in a broad sense whether switching between the native language and English produced detectable differences in neural synchrony. However, without detailed, standardized assessments of language proficiency and input, it remains difficult to determine the extent to which variability in bilingual experience may have influenced the observed patterns. Future work would benefit from incorporating more rigorous and multi-dimensional language measures.

Additionally, prior research has consistently demonstrated that interactions with familiar individuals, such as parents or romantic partners, elicit higher neural synchrony compared to strangers (Kinreich et al., 2017; Reindl et al., 2022), reinforcing the idea that emotional familiarity and relational context are key drivers of inter-brain alignment. Thus, it might be interesting to explore whether the degree of inter-brain synchrony varies depending on the language used in interactions, particularly in dyads that are less familiar or emotionally close, such as child–teacher or child–stranger pairs.

The task itself did not rely solely on verbal communication and so it would also be valuable to examine whether structured tasks, such as narrative exchange or emotional storytelling, elicit different patterns of synchrony across languages. Additionally, neural synchrony may have been supported by non-verbal behaviors such as mutual gaze, shared attention, intentionality, and the emotional bond between mother and child. These elements are central to social cognition and have been shown to elicit strong neural coupling in hyperscanning studies (Bánki et al., 2024; Lin et al., 2023; Luft et al., 2022; Markova et al., 2019; Nguyen et al., 2021a, 2023). Thus, while our study focused on the impact of language, behavioral correlates such as eye gaze, gesture, and turn-taking likely contribute to neural synchrony and should be systematically captured in future.

Our use of PTE provided a nuanced, directional measure of inter-brain connectivity. PTE is particularly well-suited for capturing non-linear and phase-based interactions and has been shown to be robust against noise and signal mixing (Lobier et al., 2014). Its application in developmental hyperscanning offers a promising avenue for understanding the dynamics of social engagement in early childhood. It is worth mentioning that even though PTE provides a sensitive and directional measure of inter-brain connectivity, permutation-based or temporally shuffled control analyses is an important addition to this study. Such null-model approaches allow for stronger inferences by confirming that observed PTE values exceed those expected from chance-level temporal alignment of the signals. While the independent-play condition offers a behavioral comparison, it does not fully substitute for a randomized control distribution. Future studies should also incorporate permutation testing or surrogate-data methods to more clearly distinguish genuine inter-brain synchrony from coincidental temporal structure in the fNIRS time series.

Last but not least, although the study involved a relatively small sample, the within-dyad repeated-measures design helps to stabilize the estimates by reducing between-participant variability and drawing on multiple observations from each dyad. Focusing the analyses on a limited number of theoretically motivated comparisons also helped ensure that the results were not driven by excessive statistical testing. Even so, the sample size remains a constraint, and the findings should be viewed as preliminary. The study offers an early contribution to understanding bilingual parent–child neural synchrony, and future research with larger samples will be important for confirming and extending these initial patterns.

### 4.2 Conclusion

This study provides novel evidence that bilingual mother–child dyads exhibit robust neural synchrony during naturalistic play, regardless of the language of interaction. By demonstrating that synchrony emerges equally in native and second-language contexts, our findings challenge the assumption that bilingualism introduces confounds to social neuroscience research. Instead, they highlight the value of ecologically valid paradigms that capture the natural dynamics of parent–child interactions. Importantly, the exploratory trends observed in relation to child-directed synchrony, child age, and maternal age suggest that developmental and contextual factors may shape the balance of engagement across dyads, warranting further investigation in larger and more diverse samples. Together, these results underscore the importance of including linguistically diverse families in developmental neuroscience. They also point toward new avenues for examining how language experience, non-verbal behaviors, and relational context contribute to inter-brain connectivity. In doing so, this work advances our understanding of the neural mechanisms supporting early bilingual social interaction and lays the groundwork for more inclusive and representative approaches to studying family dynamics in developmental science.

## Data Availability

The raw data supporting the conclusions of this article will be made available by the authors, without undue reservation.
